# Machine Learning-Based Prediction of Treatment Response to Vitamin D_3_ in Adolescents and Young Adults with Inflammatory Bowel Disease: A Prospective, Open-Label, Alternation-Based Comparative Study

**DOI:** 10.3390/biomedicines14071563

**Published:** 2026-07-12

**Authors:** Marina Adriana Mercioni, Adrian Goldiș, Nina Holban, Mihai Vasile Popescu, Radu Dragomir, Christian Goldiș, Bianca Belei, Ileana Enatescu, Roxana Folescu, Laura Olariu, Oana Belei

**Affiliations:** 1 Faculty of General Medicine, “Victor Babeș” University of Medicine and Pharmacy, 300041 Timisoara, Romania; bianca.belei@student.umft.ro; 2 Applied Electronics Department, Faculty of Electronics, Telecommunications and Information Technologies, Politehnica University Timișoara, 300223 Timisoara, Romania; mihai-v.popescu@student.upt.ro; 3 Department of Gastroenterology and Hepatology, “Victor Babeș” University of Medicine and Pharmacy, 300041 Timisoara, Romania; goldis.eugen@umft.ro; 4 Department of Computers and Information Technology, Faculty of Electrical Engineering and Computer Science, University “Stefan cel Mare” of Suceava, 720229 Suceava, Romania; ninah@seap.usv.ro; 5 Department of Obstetrics and Gynecology, “Victor Babeș” University of Medicine and Pharmacy, 300041 Timisoara, Romania; radu.dragomir@umft.ro; 6 Twelfth Department, Neonatology Clinic, “Victor Babeș” University of Medicine and Pharmacy, 300041 Timisoara, Romania; enatescu.ileana@umft.ro; 7 Department of Balneology, Medical Recovery and Rheumatology, Family Discipline, Center for Preventive Medicine, “Victor Babeș” University of Medicine and Pharmacy, 300041 Timisoara, Romania; folescu.roxana@umft.ro; 8 First Pediatric Clinic, Disturbances of Growth and Development on Children Research Center, “Victor Babeș” University of Medicine and Pharmacy, 300041 Timisoara, Romania; olariu.laura@umft.ro (L.O.); belei.oana@umft.ro (O.B.)

**Keywords:** machine learning, Crohn’s disease, ulcerative colitis, vitamin D, inflammatory bowel disease, CRP, fecal calprotectin, children, SHAP, XAI

## Abstract

**Background**: Chronic inflammatory bowel diseases (IBDs), specifically Crohn’s disease and ulcerative colitis, present substantial clinical challenges characterized by a relapsing-remitting course. These conditions often lead to serious complications and significantly impair nutritional status, physical development, and overall quality of life. **Methods**: This unicentric, prospective, open-label, alternation-based comparative study investigated the efficacy of vitamin D_3_ supplementation in adolescents with IBD. Patients aged 15–25 years were randomly assigned to receive either 1000 IU/day or 2000 IU/day of cholecalciferol for 6 months as an adjuvant to standard care. Eighty patients completed the study. **Results**: By month 6, remission was achieved in 52.5% of patients receiving 1000 IU/day (Group A) and 87.5% of those receiving 2000 IU/day (Group B). Significant differences were observed between groups regarding disease activity (*p* = 0.007), C-reactive protein levels (30.17 ± 15.74 mg/L vs. 62.82 ± 37.14 mg/L; *p* < 0.001), and fecal calprotectin levels. **Conclusions**: The machine learning models demonstrated consistent performance in assessing inflammatory indicators, with a weighted average recall exceeding 72% (Random Forest: 82.50%). No adverse effects were reported. Supplementation with 2000 IU/day of vitamin D_3_ for 6 months was associated with more favorable clinical and biochemical outcomes than 1000 IU/day in this single-center open-label study as an adjunct to standard treatment in adolescents and young adults with IBD. The results suggest that higher doses of vitamin D may be beneficial in achieving remission and reducing systemic inflammation.

## 1. Introduction

Chronic inflammatory bowel diseases (IBDs), which include Crohn’s disease (CD) and ulcerative colitis (UC), pose significant healthcare challenges due to their relapsing-remitting nature, risk of complications, and significant impact on growth, nutrition, and quality of life, especially when onset occurs in childhood or adolescence [1]. The burden of pediatric-onset IBD is exacerbated by concerns such as growth retardation, delayed puberty, reduced bone mineralization, and long-term health effects. For example, a recent cohort discovered that vitamin D insufficiency and low bone mineral density (BMD) were prevalent endocrine problems in children and adolescents with IBD [2].

There are relatively few pediatric studies focused on the potential benefit of vitamin D supplementation on the clinical course of IBD in adolescents and young adults.

Recent studies have investigated the role of vitamin D in maintaining optimal bone health and reducing inflammation in children IBD. Targeting serum 25-hydroxyvitamin D (25OHD) concentrations ≥ 30 ng/mL is recommended for this population.

Vitamin D affects calcium and phosphate balance, and hence skeletal integrity. However, in recent years, more emphasis has been directed to its extra-skeletal functions, including immune response regulation, epithelial barrier integrity maintenance, and effect on the gut microbiome. These functions may connect vitamin D to the etiology and progression of IBD. Vitamin D receptor (VDR) signaling in intestinal epithelial cells, for example, has been linked to inflammation control, regulation of antimicrobial peptide expression, modification of T-cell subsets, and modulation of gut barrier tight junctions [3].

Considering these molecular relationships, it is physiologically possible that vitamin D levels may affect the start of IBD, its severity, and treatment response; conversely, IBD may lead to vitamin D insufficiency due to diminished sun exposure, malabsorption, inflammation, and altered metabolism. Diminished sun exposure can be due to frequent hospitalizations, chronic fatigue, or reluctance to leave the house because of gastrointestinal symptoms [4]. Observational studies suggest that vitamin D insufficiency or inadequacy is prevalent among adolescents and young adults with IBD.

Observational studies reveal that vitamin D insufficiency/deficiency is frequent among adolescents and young adults with IBD [5]. In another pediatric study, vitamin D status was strongly associated with lumbar spine BMD. In a retrospective observational study involving children and adolescents with IBD, vitamin D deficiency—defined as 25(OH)D_3_ levels below 20 ng/mL—was exceptionally prevalent, being documented in 81.7% of the evaluated patients (94 out of 115) with an average level of 14.5 ± 7.0 ng/mL. Furthermore, the study demonstrated a significant positive correlation between serum 25(OH)D_3_ levels and BMD Z-scores (r = 0.215, *p* = 0.024). Among the cohort, 21.0% of patients presented with osteoporosis (lumbar BMD Z-score ≤ −2.0), and these specific patients exhibited low mean vitamin D levels of 14.01 ± 5.71 ng/mL, underscoring the strong association between poor vitamin D status and impaired bone health in pediatric IBD [2].

Therefore, although insufficiency is widespread, it is still unclear how strongly low vitamin D is associated with IBD activity or severity.

The objective of interventional trials examining vitamin D supplementation in children with IBD has been to determine whether correcting vitamin D deficiency improves clinical and biochemical outcomes. For example, a randomized study found that administering either 2000 IU of vitamin D_3_ daily or 50,000 IU of vitamin D_2_ weekly was significantly more effective at increasing 25(OH)D levels than giving 2000 IU of vitamin D_2_ daily. Both regimens showed excellent tolerability in adolescents and young adults with IBD and 25(OH)D levels below 20 ng/mL [6].

A study involving youth aged 5 to 21 found that even taking 2000 IU of vitamin D daily was insufficient to maintain a vitamin D level of 32 ng/mL throughout the year. This trial compared three approaches: daily doses of 2000 IU of vitamin D_3_, daily doses of 2000 IU of vitamin D_2_, and 50,000 IU D_2_ weekly. The study revealed that supplementation with 2000 IU/day vitamin D_3_ or 50,000 IU/week vitamin D_2_ was more effective than daily administration of 2000 IU vitamin D_2_ for six weeks in increasing serum 25(OH)D levels among young patients suffering from IBD and vitamin D deficiency. Notably, the group receiving higher doses experienced fewer instances of elevated C-reactive protein (CRP) levels [6].

Research reviews in pediatric IBD have demonstrated positive impacts of supplementation on 25(OH)D levels, along with a decrease in inflammatory markers like CRP and erythrocyte sedimentation rate (ESR) in adolescents and young adults undergoing vitamin D treatment, especially at doses exceeding 2000 IU and with follow-up periods longer than 12 weeks [7].

Despite the promising findings, significant gaps persist in the research. Most studies concentrate on correcting vitamin D levels rather than examining critical clinical outcomes such as relapse rates and mucosal healing. Furthermore, there is no agreement on the optimal target for 25(OH)D levels in pediatric IBD, nor is there a standardized regimen for dosage and duration that consistently achieves and maintains these targets. Additionally, few studies incorporate disease-specific measures—such as disease extent, activity indices, and biomarkers—to evaluate how changes in vitamin D correlate with responses to IBD treatment. The variability and often limited responses in 25(OH)D levels among IBD patients, when compared to healthy individuals, indicate that various disease-related factors, including malabsorption, inflammation, and treatments, may disrupt vitamin D metabolism.

Additionally, puberty is a crucial time for long-term prognosis formation, the establishment of health habits (diet, sun exposure), and the transition of care. Therefore, maintaining normal vitamin D levels may assist long-term bone health, extra-intestinal issues, and skeletal results in addition to acute illness treatment.

The clinical response to vitamin D administration varies, despite the substantial molecular plausibility of vitamin D in modifying IBD. Results may be impacted by variations in baseline vitamin D status, illness phenotype (extent, severity), therapy (immunomodulators, biologics), biomarker levels (CRP, fecal calprotectin), growth/pubertal status, and other factors. Complex, non-linear interactions and temporal trajectories (e.g., how change in 25(OH)D from T0 to T1 interacts with baseline illness score, or how therapy type changes the effect of vitamin D change) may be difficult for traditional statistical analyses to detect. A ML approach provides a potent solution for deciphering the intricate interactions between vitamin D_3_ supplementation, disease activity, and inflammatory biomarkers. Through the use of high-dimensional longitudinal data, such as demographics, treatment plans, Paris classification, and serial measurements of 25(OH)D, CRP, fecal calprotectin, and clinical scores at various time points ML algorithms are able to identify patterns that are not easily discernible using conventional statistical techniques.

The efficacy of vitamin D supplementation in adolescents and young adults with IBD has been investigated, alongside the development of algorithms to predict disease severity (remission to severe).

This study aimed to determine whether a dose of 1000 IU or 2000 IU of orally administered vitamin D can effectively reduce intestinal inflammation and improve clinical scores at 3 and 6 months. The relationship between vitamin D intake, disease activity, and inflammatory markers was also assessed after administration of two different dosage regimens.

The algorithms developed in this study aimed to predict the severity of disease remission in patients six months later (T2), based on their initial profile. This predictive model sought to forecast whether a patient would experience remission, mild, moderate, or severe disease activity at T2.

## 2. Materials and Methods

### 2.1. Study Design and Population

**Figure 1:**
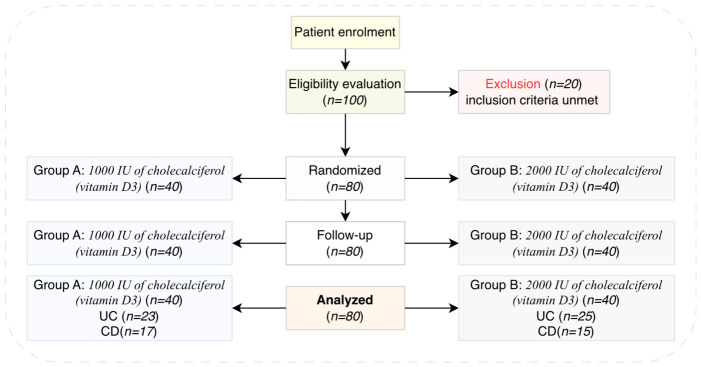
Adolescents and young adults with IBD enrolment algorithm.

A single-center, prospective, open-label, alternation-based comparative study was conducted to evaluate the efficacy of oral vitamin D supplementation in adolescents and young adults with IBD, specifically UC and CD (Figure 1).

**Figure 2:**
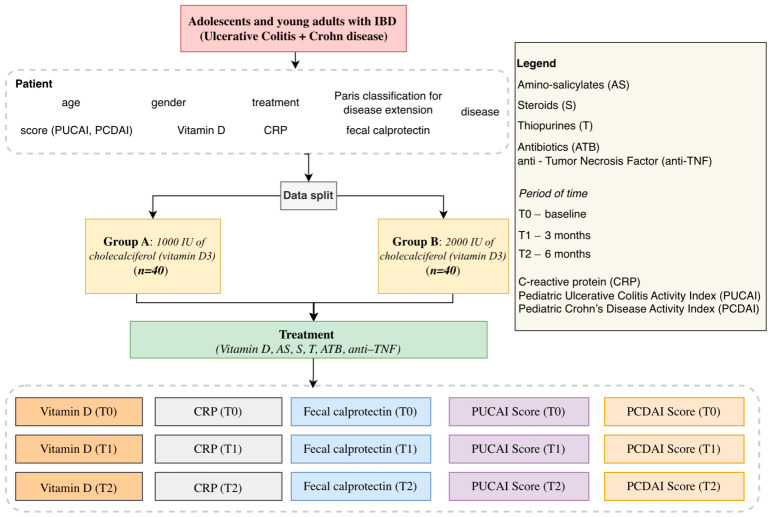
Adolescents and young adults with IBD—study design.

Figure 2 illustrates the experimental design of the study focusing on adolescents and young adults with IBD, specifically including patients with UC and CD. The methodology involves a cohort split into two distinct groups (n = 40 per group) to evaluate the efficacy of different daily dosages of cholecalciferol (vitamin D_3_): 1000 IU for Group A and 2000 IU for Group B. Throughout the six-month study period, vitamin D supplementation is administered alongside standard treatments such as aminosalicylates, steroids, thiopurines, antibiotics and anti-TNF therapies. The longitudinal impact is assessed at three intervals—baseline (T0), three months (T1), and six months (T2)—by monitoring key clinical and biochemical markers, including serum vitamin D levels, CRP, fecal calprotectin, and disease activity indices (PUCAI and PCDAI).

### 2.2. Inclusion Criteria

- Age: Patients aged 15–25 years.
- Previous diagnosis of Inflammatory Bowel Disease (IBD) with current remission at time of enrollment.
- Undergoing standard maintenance treatment for IBD, as per national guidelines.
- Serum 25-Hydroxyvitamin D level < 30 ng/mL at baseline, indicating hypovitaminosis D.

### 2.3. Exclusion Criteria

- Presence of chronic liver or kidney disease.
- Receipt of systemic corticosteroid therapy within 2 months prior to enrollment.

Due to universal hypovitaminosis D among study participants, vitamin D supplementation was a mandatory requirement for all enrolled patients.

The selection of 1000 IU and 2000 IU vitamin D doses for adolescents was based on pharmacological rationale. A daily dose of 2000 IU approaches a therapeutic range, potentially exceeding simple supplementation levels.

This study utilized an open-label design; consequently, neither participants nor investigators were blinded to the treatment assignments. Participants were enrolled consecutively upon diagnosis and assigned to treatment groups (1000 IU or 2000 IU) in an alternating sequence, ensuring a systematic distribution based on the order of presentation. The study enrolled 80 adolescents and young adults aged 15–25 years diagnosed with IBD in clinical remission. Patients meeting the inclusion criteria were open-label, alternation-based to receive either 1000 IU/day of cholecalciferol (vitamin D_3_) (Group A) or 2000 IU/day of vitamin D_3_ (Group B), both administered over the study period (baseline, 3 months, 6 months). This design enabled a controlled comparison of dose-dependent effects of vitamin D_3_ supplementation on serum 25(OH)D trajectories, inflammatory biomarkers, and disease activity outcomes in adolescents and young adults with IBD.

### 2.4. Endpoints

The interest endpoints of the study were:

- Relapse rate: the proportion of patients experiencing a relapse, which was a key outcome measure to assess treatment effectiveness.
- Vitamin D response: assessed at baseline and after 3 and 6 months of treatment.
- CRP response: evaluated at baseline and after 3 and 6 months of treatment.
- Fecal calprotectin levels: measured at baseline, 3 months, and 6 months post-treatment.
- Pediatric UC Activity Index (PUCAI) and Pediatric CD Activity Index (PCDAI) scores: assessed at baseline, 3 months, and 6 months post-treatment.

### 2.5. Statistical Analysis

The statistical analysis of this study aimed to evaluate the effectiveness of different treatment types in adolescent and young adult patients with IBD. To accomplish this, rigorous statistical methods tailored to the analyzed data were applied.

#### 2.5.1. Descriptive Analysis (Baseline T0)

The baseline characteristics of the study population were analyzed to provide a comprehensive understanding of the patients’ demographic and clinical profiles at the start of the treatment.

Comparison of the two treatment groups at T0 was performed to verify comparability (to ensure no significant differences existed prior to treatment).

Statistical tests were applied as follows:

- *t*-test for normally distributed variables: age, vitamin D levels.
- Mann–Whitney U test for non-normally distributed scores and inflammatory markers: CRP and calprotectin.

To compare groups for continuous variables such as age, PUCAI, PCDAI scores, fecal calprotectin, and CRP, an independent samples *t*-test was used. The findings were reported using *p*-values to evaluate statistical significance and were presented as mean ± standard deviation (SD). Using the Chi-square test, relationships were examined for categorical factors including gender, disease, Paris classification for disease extension, disease severity, and treatment regimens (e.g., the use of amino-salicylates, steroids, thiopurines, antibiotics, or anti-TNF).

Changes in PUCAI, PCDAI scores (Table 1), CRP, and fecal calprotectin were analyzed across multiple time points (PUCAI, PCDAI at baseline and after 3 and 6 months of treatment, and CRP, fecal calprotectin at baseline and after 3 and 6 months of treatment).

We determined the serum 25(OH)D thresholds based on laboratory references: <20 ng/mL indicates vitamin D deficiency; 20–30 ng/mL signifies vitamin D insufficiency; and >30 ng/mL denotes vitamin D sufficiency [8].

These analyses primarily relied on the Independent Samples *t*-test and Paired Samples *t*-test. Results were reported in their respective units, such as CRP (normal reference range < 5.0 mg/L) and fecal calprotectin (normal reference range < 50 µg/g) (Table 2), with a threshold for statistical significance set at *p* < 0.05.

**Table 1:** Classification of disease severity (activity).

	Score	Severity	Status
PUCAI *	0–10	Remission	Inactive
	10–34	Mild	Active
	35–64	Moderate	Active
	65–85	Severe	Active
PCDAI *	6.8±6.6	Remission	Inactive
	18.7±7.3	Mild	Active
	38.5±12.9	Moderate	Active
	54.2±14.0	Severe	Active

* Pediatric UC Activity Index (PUCAI) [9] and Pediatric CD Activity Index (PCDAI) [10] scores.

**Table 2:** Inflammatory markers.

	Score	Severity
CRP * (mg/L)	<5.0	Normal, no significant inflammation
	5–10	Mild inflammation, may suggest mild IBD activity
	>10	Moderate to severe inflammation, commonly seen in active IBD
	>50	Severe inflammation, may indicate a flare or complication
Fecal calprotectin (µg/g)	<50	Normal, no significant inflammation
	≤200	Borderline, mild inflammation
	≤500	Moderate inflammation, suggestive of active IBD
	>500	High inflammation, indicative of active IBD or flare

* CRP (C-reactive protein Pediatric).

#### 2.5.2. Longitudinal Analysis (Evolution from T0 to T1 to T2)

The study population was followed over time, and changes in the variables of interest were analyzed across three time points: baseline (T0), 3 months post-treatment (T1), and 6 months post-treatment (T2) using Mixed ANOVA and Linear Mixed Models (LMM).

#### 2.5.3. Correlation Analysis

A correlation analysis was performed to investigate the associations between vitamin D levels and Calprotectin concentrations, as well as CRP values. Specifically, Spearman rank-order correlations were calculated to examine the relationships between these variables, while Pearson product-moment correlations were also computed for comparative purposes.

Finally, the study conducted additional analyses to evaluate the predictive value of CRP and fecal calprotectin thresholds. Receiver operating characteristic (ROC) curve analysis was used to determine sensitivity, specificity, and the area under the curve (AUC) for baseline CRP and fecal calprotectin as prognostic markers. Correlation analyses further explored the relationship between PUCAI, PCDAI scores and clinical outcomes, including treatment response, CRP, and fecal calprotectin.

### 2.6. ML Approach

The ML methodology adhered to a systematic workflow originating from a raw CSV dataset. Data cleaning and preparation were carried out by using StandardScaler (https://scikit-learn.org/stable/, accessed on 4 July 2026) data preprocessing method to standardize numerical features, while categorical variables were encoded one-hot. Logistic Regression, and Random Forest classifiers were used as models. The model’s performance was evaluated using stratified 5-fold cross-validation, using accuracy, precision, recall, F1-score as assessment measures. In a 5-fold stratified cross-validation procedure, an 80-sample dataset was divided equally into five subsets. In each iteration (fold), four of these subsets (64 samples) were used for model training, while the remaining subset (16 samples) served as the test set. This approach ensured that every sample in the original dataset was utilized once in the test set and four times in the training set, maintaining class proportions within each fold to provide a robust evaluation of the model’s performance. The sensitivity analysis was conducted to evaluate the robustness of the Random Forest and Logistic Regression models to changes in their key hyperparameters. For Random Forest, the n_estimators parameter was varied, while for Logistic Regression, the *C parameter* (inverse of regularization strength) was adjusted. Model performance was assessed using the F1-macro score, obtained through a 5-fold Stratified K-Fold cross-validation strategy. This approach allowed for an examination of how each model’s predictive ability changed across a range of parameter values, while keeping other hyperparameters constant.

**Figure 3:**
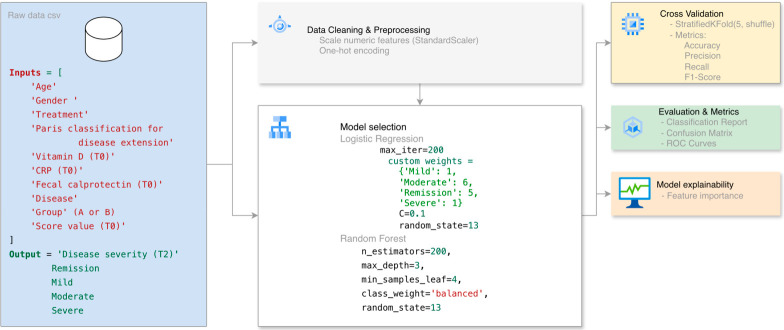
ML—study design.

A classification report, confusion matrix, and ROC curve analysis were used in the final evaluation, and model explainability for feature importance analysis (Figure 3). This work was developed using Python version 3.12.13, scikit-learn version 1.6.1 (https://scikit-learn.org/stable/), pandas version 2.2.2 (https://pandas.pydata.org/), numpy version 2.0.2 (https://numpy.org/), scipy version 1.16.3 (https://scipy.org/), matplotlib version 3.10.0 (https://matplotlib.org/) in a Google Colaboratory environment for setting up and training the models.

The features were manually selected at T0, T1 and T2 to include ‘Age’, ‘Gender’, ‘Treatment’, ‘Paris classification’, ‘Vitamin D’, ‘CRP’, ‘Fecal calprotectin’, ‘Disease’, ‘Group’ and ‘Score value’. Data preprocessing was managed using a ColumnTransformer and Pipeline (https://scikit-learn.org/stable/, accessed on 4 July 2026) to prevent data leakage between training and testing folds. Missing values were imputed first, followed by scaling and one-hot encoding. StandardScaler was applied to numeric features, while OneHotEncoder (https://scikit-learn.org/stable/, accessed on 4 July 2026) was used for categorical features. These preprocessing steps being incorporated into a Pipeline meant that the fit (StandardScaler, OneHotEncoder, SimpleImputer [https://scikit-learn.org/stable/, accessed on 4 July 2026]) occurred only on training data of each fold, with transform then applied to both training and testing data in that fold. This ensured that the model did not “see” information from the test set during preprocessing, maintaining the rigor of cross-validation. Missing values were handled by imputation using SimpleImputer within the pipeline. For numeric features, strategy = ‘median’ was used. For categorical features, strategy = ‘most_frequent’ was employed. A sensitivity analysis was performed to explore the impact of primary hyperparameters on model performance. This is a form of exploring the hyperparameter space. For Random Forest, parameter n_estimators (number of trees in forest) was varied over [50, 100, 200, 300, 400, 500]. For Logistic Regression, parameter C (inverse power regularization strength) was varied over [0.001, 0.01, 0.1, 1, 10, 100].

Results were visualized to identify trends and potential optimal values of these hyperparameters based on the F1-macro median score obtained through cross-validation.

To ensure reproducibility of results, a random_state was set for all components involving randomness. StratifiedKFold(random_state = 42) was used for cross-validation. LogisticRegression(random_state = 13) and RandomForestClassifier(random_state = 13) were used for logistic regression and Random Forest models, respectively. Models were evaluated directly based on predicted probabilities and class predictions. Explainable AI (XAI) allows us to understand, interpret, and explain the decisions made by ML models. SHapley Additive exPlanations (SHAP) is used to interpret the ML models’ predictions.

## 3. Results

The study included 80 participants, evenly divided into two treatment groups (N = 40 per group): Group A received a daily dose of 1000 IU of cholecalciferol, while Group B received 2000 IU. Table 3 confirms the fundamental validity of the clinical trial by illustrating the baseline homogeneity of the study cohorts before the therapeutic intervention. All resulting variations in clinical outcomes may be statistically linked to variations in vitamin D_3_ intake rather than pre-existing structural imbalances across the groups, due to the equal distribution and thorough reporting of baseline data. The cohort’s mean age, which is around 19 years old, 19.27 ± 3.03 in Group A and 19.07 ± 3.03 in Group B, places the individuals in a crucial biological window: the change from late adolescence to early adulthood. To reach maximal bone mass and stabilize the immune response, this time is physiologically crucial. Additionally, the gender distribution is almost symmetrical, which eliminates any confounding factors pertaining to hormonal impacts or gender-specific vitamin D metabolism. In terms of the clinical profile of IBD, the results show that UC is more common in both groups (~60%) than CD (~40%). Validated clinical tools, namely the PUCAI for UC and the PCDAI for CD, were used to track disease activity. The study’s focus on a population with complicated phenotypes and extensive intestinal involvement is highlighted by the categorization of patients based on these scores and the Paris Classification for illness extension, which notes a considerable incidence of E4 (pancolitis) and L2/L3 localizations. The variety of treatment plans used, with most patients receiving combination therapy that includes aminosalicylates, corticosteroids, and biological or immunosuppressive drugs, further illustrates the complexity of clinical care. The clinical reality of young IBD patients, who frequently need intensive therapy to maintain inflammatory control, is reflected in this variability. The claim that concomitant drug therapy will not impact results is supported by the fact that these drugs are equally divided across Group A and Group B. Regarding concomitant IBD treatments, all participants were maintained on their established therapeutic regimens throughout the study period. At baseline (T0), there were no significant differences between Group A and Group B in the use of corticosteroids, antibiotics, immunomodulators, or biological therapies (*p* > 0.05). To ensure the internal validity of the findings, these treatments remained stable during follow-up, with no dose adjustments or new therapy inductions permitted. This stability across both cohorts suggests that the observed improvements in clinical and biochemical outcomes were primarily associated with the vitamin D_3_ intervention rather than changes in background medical therapy.

The groups were found to be comparable (no statistically significant differences) in terms of age, vitamin D levels, CRP, fecal calprotectin, and score value at baseline (Table 4).

**Figure 4:**
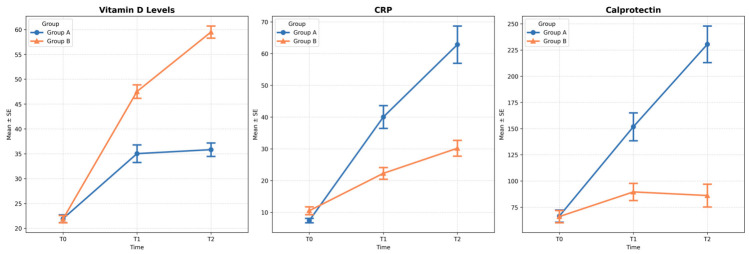
Comparison of Treatment Groups A and B: the levels of vitamin D, CRP, and fecal calprotectin were compared between groups A and B at three time points: baseline (T0), 3 months post-treatment (T1), and 6 months post-treatment (T2).

The statistical analysis results indicate significant effects of time and interaction between groups on all three variables: vitamin D, CRP, and calprotectin. Specifically, the effect of time is strongest for vitamin D ($\eta_{p}^{2}$ = 0.768), while the interaction between groups has a significant impact on all three variables (Table 5). These findings suggest that treatment had different effects on the two patient groups and that levels of vitamin D, CRP, and calprotectin evolved differently over time (Figure 4).

The results indicate significant interactions between time and group for all three variables: vitamin D Levels, CRP, and Calprotectin. Specifically, the coefficients reveal that Group B exhibits a more pronounced increase in vitamin D Levels at both Time 1 (12.57) and Time 2 (23.75), compared to Group A. Conversely, Group B shows a greater decrease in CRP levels at both time points (−20.85 and −35.75). Similarly, the coefficients for Calprotectin indicate that Group B experiences a more significant decline in this variable over time (−61.75 and −144.00), compared to Group A (Table 6).

**Figure 5:**
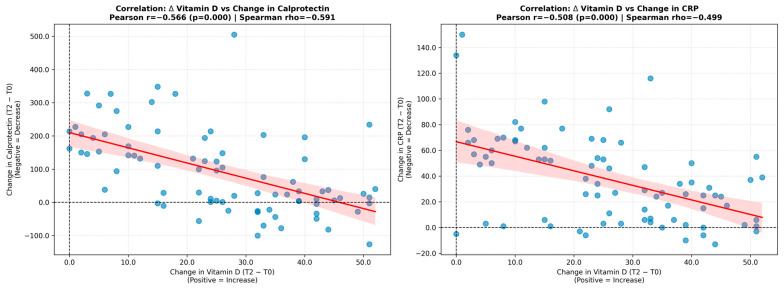
Correlation between Change in Vitamin D and Changes in Inflammatory Markers. These plots illustrate the bivariate correlations between the change in Vitamin D levels (T2–T0) and the changes in two key inflammatory markers: Fecal Calprotectin (**left**) and CRP (**right**). Each point represents an individual patient, with the x-axis indicating the change in Vitamin D (positive values denote an increase, negative a decrease) and the y-axis showing the change in the respective inflammatory marker (positive values denote an increase, negative a decrease). A linear regression line (red) with a 95% confidence interval (shaded red area) is overlaid on each scatter plot. Both figures display a negative correlation, where an increase in Vitamin D levels tends to be associated with a decrease in Fecal Calprotectin (Pearson r = −0.566, *p* < 0.001) and CRP (Pearson r = −0.508, *p* < 0.001). Spearman’s rho also indicates significant negative rank correlations (Fecal Calprotectin: rho = −0.591, *p* < 0.001; CRP: rho = −0.499, *p* < 0.001). Quadrant lines at (0,0) divide the plots to help visualize patterns of change.

A significant inverse correlation was observed between the increase in serum vitamin D levels and the reduction in Fecal Calprotectin concentrations. Specifically, as vitamin D levels increased, there was a corresponding decrease in Calprotectin values. This association was statistically confirmed by both Pearson (r = −0.566) and Spearman (rho = −0.591) correlation analyses, indicating a moderate to strong negative relationship between these variables. Similarly, a significant inverse correlation was also observed between the increase in serum vitamin D levels and CRP concentrations. The Pearson correlation analysis revealed a weak to moderate negative association (r = −0.508), while the non-parametric Spearman test confirmed this finding with a rho value of −0.499, both at *p* < 0.001 (Figure 5).

Table 7 shows that the distribution of disease severity differs significantly between the two research arms, indicating a higher remission profile within Group B. Group A (1000 IU) had remission rates of around 47% for CD (8 out of 17 patients) and 56.5% for UC (13 out of 23 patients), with a significant number of individuals continuing demonstrating moderate disease activity (8 CD cases and 9 UC cases). Group B (2000 IU) had much better clinical outcomes, with the great majority of patients in remission: 86.6% for CD (13 out of 15 patients) and 88% for UC (22 out of 25 patients). Furthermore, intermediate disease cases were much fewer in Group B (2 CD and 3 UC cases), whereas severe cases were completely missing, in contrast to Group A, which had one case of severe UC recorded. These findings imply a link between increased vitamin D_3_ dosages and the maintenance of clinical remission in most of the patients in the analyzed cohort.

Post-treatment results presented in Table 8 indicate that a significantly higher proportion of participants in Group B achieved clinical remission (87.5%) compared to Group A (52.5%). Moreover, the prevalence of moderate-to-severe disease activity was markedly lower in the 2000 IU group (12.5%) than in the 1000 IU group (45.0%), with no severe cases reported in the higher-dosage cohort. A between-group statistical comparison confirmed these differences were significant, with a reported *p*-value of 0.007, satisfying the criteria for statistical significance (*p* < 0.05).

High-dose vitamin D_3_ intake (2000 IU) resulted in considerably greater serum levels at 6 months (59.50 ± 7.73 ng/mL) than the 1000 IU dose (35.82 ± 8.5 ng/mL), which was associated with significantly better clinical and biochemical stability (Table 9). At the six-month follow-up, Group B had significantly lower inflammatory marker levels, with CRP at 30.17 ± 15.74 µg/g and fecal calprotectin at 86.10 ± 68.90 µg/g, whereas Group A had significant increases in both parameters.

**Figure 6:**
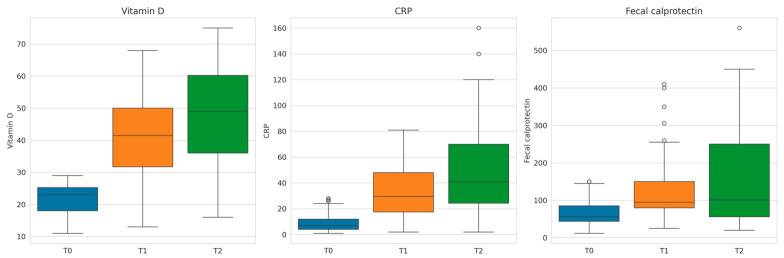
Distribution of Key Biomarkers Across Timepoints (T0, T1, T2). These box plots illustrate the median, interquartile range (IQR), and potential outliers for Vitamin D levels, CRP, and Fecal Calprotectin at baseline (T0), after 3 months (T1), and 6 months (T2) timepoints. The central line in each box indicates the median, the box boundaries represent the 25th and 75th percentiles (IQR), and the outliers. This visualization provides insights into the spread and central tendency of each biomarker’s values at different stages of the study, allowing for a qualitative assessment of changes over time within the overall study population.

The longitudinal analysis shown in Figure 6 shows a consistent and substantial rise in serum 25(OH) vitamin D levels throughout the research period, with the median increasing from roughly 22 ng/mL at baseline (T0) to 50 ng/mL at the last six-month evaluation (T2). In contrast to optimizing vitamin D status, inflammatory markers—specifically CRP and fecal calprotectin—show an upward trend in median values and a significantly wider distribution of data towards the end of the monitoring period. Extreme outliers reach 160 mg/L for CRP and 550 µg/g for fecal calprotectin. These visual illustrations corroborate the successful treatment of vitamin D deficiency across the sample while also indicating a persisting, diverse, and significant inflammatory response in certain individuals despite therapeutic supplementation.

The Random Forest model demonstrated superior performance compared to Logistic Regression, with a weighted average recall of 0.8250 and accuracy rate of 82.5%, indicating that approximately 83% of patients were correctly classified as having achieved remission or being at a specific level of disease severity (mild, moderate, severe). Notably, the model’s precision in predicting remission was also high, with an F1-score of 0.8793 and recall rate of 0.9107, suggesting its potential utility in identifying patients who have responded favorably to treatment (Table 10).

The Odds Ratios (ORs) Table 11 presents the change in odds of disease severity status (Mild, Moderate, Remission) associated with a one-unit increase in each feature. The reference category is “Severe” disease severity. An OR greater than 1 indicates an increased likelihood of being classified as “Compared Class” (Remission, Mild, Moderate), whereas an OR less than 1 signifies a decreased likelihood relative to the “Reference Class” (Severe). An OR equal to 1 implies no change in odds. For instance, examining the row for vitamin D (T0), an Odds Ratio of 0.9068 for Mild indicates that each one-unit increase is associated with approximately a 9% decrease in the likelihood of having Mild disease severity compared to Severe. Conversely, an OR of 1.6355 for Moderate suggests that every one-unit increase corresponds to about a 64% higher odds ratio of being classified as Moderate versus Severe.

**Figure 7:**
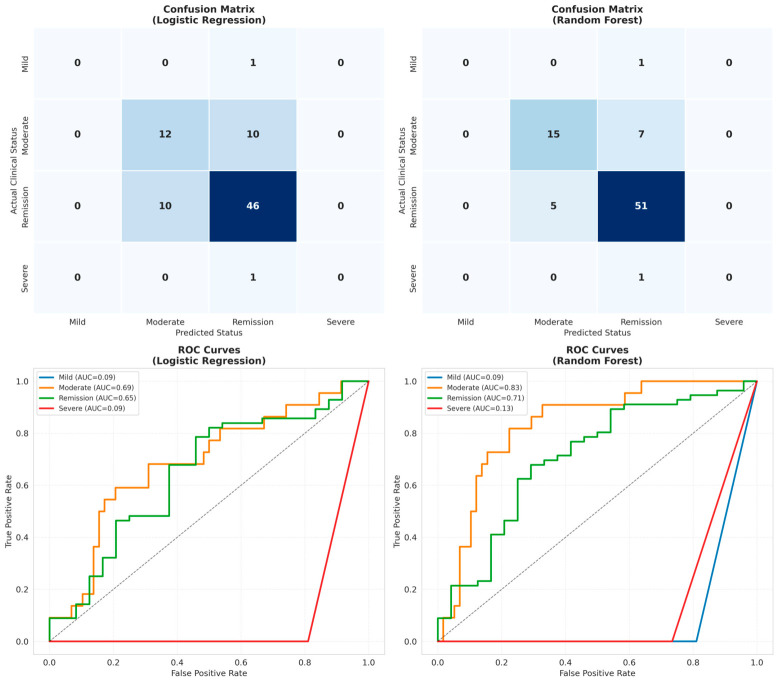
Combined Classification Performance of Logistic Regression and Random Forest Models. This figure presents a two-panel visualization of the classification performance for the Logistic Regression and Random Forest models across different disease severity categories. The top row displays Confusion Matrices for each model, illustrating the count of correctly and incorrectly classified instances for each class (Mild, Moderate, Remission, Severe). The bottom row shows the ROC Curves for each model, with corresponding AUC scores for each severity class. The ROC curves and AUC values provide insights into the models’ ability to discriminate between positive and negative classes at various threshold settings. The AUC for each ROC curve quantifies the overall performance of the model across all possible classification thresholds; a higher AUC value (closer to 1.0) indicates better separability between the classes and thus better model performance. The dotted line represents the ROC curve for a random classification. Each model was trained and evaluated using 5-fold stratified cross-validation.

The confusion matrix and ROC curve analysis revealed that the Random Forest model demonstrated exceptional discriminatory power in distinguishing between patients with different disease severities, particularly those in remission (AUC = 0.71), moderate severity (AUC = 0.83) and mild/severe categories (AUCs of 0.09 and 0.13, respectively). Notwithstanding the challenges posed by an unbalanced dataset for the latter two classes, these results indicate a high level of accuracy in classifying patients into their respective disease severity categories (Figure 7).

**Figure 8:**
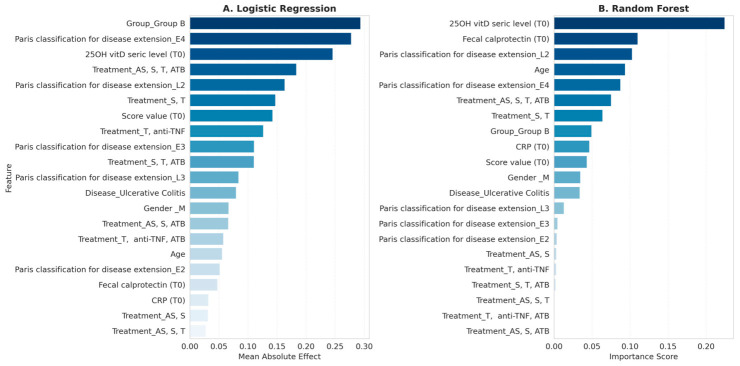
Feature importance.

The feature importance analysis revealed that the top three features contributing to the predictive power of the Logistic Regression model were Group B (2000 UI vitamin dosage), Paris classification for disease extension (E4), vitamin D level at baseline (T0), and Random Forest’s most important features being vitamin D level at baseline (T0), Fecal calprotectin levels at baseline (T0), and Paris classification for disease extension (L2). These findings suggest that the models leverage a combination of clinical, biochemical, and endoscopic markers to predict disease severity (Figure 8).

**Figure 9:**
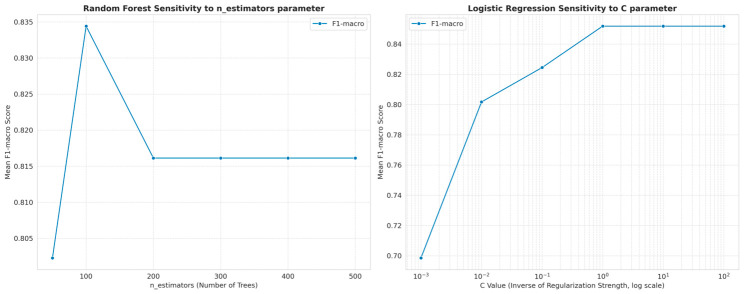
The sensitivity analysis. Random Forest and Logistic Regression models.

The sensitivity analysis indicates that for the Random Forest model, the F1-macro score peaked around n_estimators = 100, with diminishing returns or slight decreases in performance beyond this point. For Logistic Regression, the F1-macro score generally improved with increasing C_Value (lower regularization strength), reaching a plateau (Figure 9).

**Figure 10:**
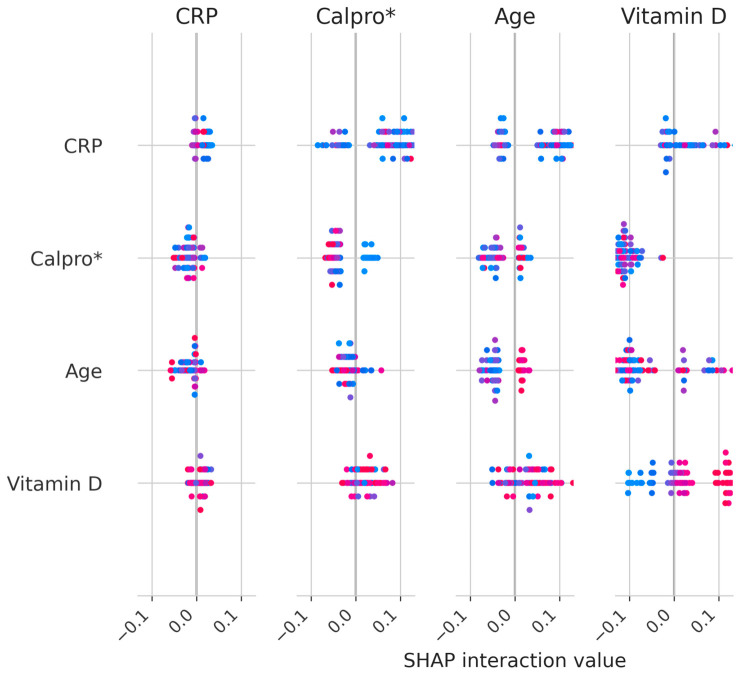
Explainable AI. The SHAP interaction plot reveals how combinations of features influence the model’s prediction of disease severity, Calpro *—Fecal Calprotectin; Vitamin D and CRP at baseline (T0). Each row represents a feature being analyzed for its main effect, while each column corresponds to an interacting feature. The horizontal axis of the scatter plot displays the SHAP interaction value for a specific instance. Points are colored according to the interacting feature’s value (red for high values and blue for low). Positive interaction values indicate that the combined influence of both features enhances the prediction in a particular direction, suggesting synergy. Conversely, negative values suggest an antagonistic effect.

The SHAP interaction plot (Figure 10) reveals how combinations of features influence the model’s prediction of disease severity. For instance, high baseline CRP (red dots in the CRP(T0) row, CRP(T0) column) and high Fecal Calprotectin (red dots in the Fecal Calprotectin (T0) row, Fecal Calprotectin (T0) column) individually contribute positively to the prediction of higher severity, indicated by larger SHAP interaction values. Notably, lower Vitamin D seric levels at T0 (blue dots in the Vitamin D seric level (T0) row, Vitamin D seric level (T0) column) also show a tendency towards positive SHAP interaction values, suggesting that a deficiency in vitamin D at baseline is associated with predictions of increased severity. Furthermore, the plot highlights interactions, such as between CRP(T0) and Fecal Calprotectin(T0), where high values of both features appear to amplify the positive impact on the model’s output, indicating a synergistic effect in predicting disease severity.

Safety was rigorously assessed by monitoring serum calcium, urinary calcium, and renal function at baseline and at baseline (T0), 3 months post-treatment (T1), and 6 months post-treatment (T2); all parameters remained within normal physiological limits throughout the study. As detailed in Table 12, mean serum calcium and 24 h urinary calcium levels remained within physiological reference ranges (8.5–10.5 mg/dL and 100–300 mg/24 h, respectively) throughout the study for both the 1000 IU and 2000 IU groups. Renal function, assessed via serum creatinine and eGFR, showed no clinically significant fluctuations, with all mean values maintaining an eGFR ≥ 90 mL/min/1.73 m^2^.

No instances of hypercalcemia, hypercalciuria, or renal abnormalities were observed in either group during the follow-up period. Furthermore, the intervention was well-tolerated; there were no reported gastrointestinal symptoms or serious adverse events, and no participants discontinued the study due to safety concerns. These findings support the conclusion that a daily dose of 2000 IU vitamin D_3_ is associated with a favorable safety profile in this population.

## 4. Discussion

The main results of the study are summarized in this chapter, emphasizing the 2000 IU vitamin D_3_ dose’s superiority in the clinical and biochemical therapy of patients with inflammatory bowel disease (IBD) who are adolescents or young adults.

The findings show a distinct dose–response relationship, with 2000 IU of cholecalciferol treatment being substantially more successful than 1000 IU in raising serum 25(OH)D levels. Significantly greater clinical remission rates exceeding 86% for both IBD types were clearly connected with this better rise in Group B (59.50 ± 7.73 ng/mL vs. 35.82 ± 8.51 ng/mL in Group A). Group A, on the other hand, had prolonged moderate disease activity, suggesting that typical 1000 IU dosages might not be enough for total symptom management in this age range [11,12,13,14].

The immunomodulatory function of vitamin D in the intestinal mucosa is supported by the found negative connection between vitamin D levels and inflammatory markers (CRP: r = −0.50; fecal calprotectin: r = −0.56). After six months, Group B’s fecal calprotectin levels dramatically decreased (86.10 µg/g) compared to Group A’s high levels (230.55 µg/g), suggesting that local inflammation management is linked to adequate vitamin status. These results are crucial for young patients (mean age ~19 years), since subclinical inflammation reduction promotes bone mineral density consolidation and avoids long-term problems [15,16].

These findings have critical therapeutic implications for the population of adolescents and young adults. Optimizing vitamin D levels is an essential preventative measure since these individuals are more likely to have bone demineralization as a result of long-term corticosteroid treatment and chronic inflammation [17]. Furthermore, the immunomodulatory function of vitamin D is critical for sustaining clinical remission, which is necessary for young people [18].

The integration of machine learning algorithm insights into the study’s conclusions underscores the potential for personalized monitoring in adolescent and young adult IBD patients.

Given the small sample size (*n* = 80) and tabular nature of the data, traditional ML models such as Logistic Regression and Random Forest were preferred over Artificial Neural Networks (ANNs) [19]. This decision was based on practical and statistical considerations. Specifically, ANNs typically require a large amount of data to train their numerous parameters effectively and generalize well, which is not feasible with only 80 samples, leading to potential overfitting and poor performance on new data. In contrast, Logistic Regression and Random Forest offer more interpretable results regarding variable importance and relationships, crucial in medical contexts for understanding risk factors or prognostic indicators.

Notably, Random Forest emerged as the most reliable method, achieving precision, recall, and F1-score values of 0.8500, 0.9107, and 0.8793, respectively. These results surpassed previous research (accuracy of 0.77) [20]. The feature importance analysis revealed that Group B (2000 UI vitamin dosage), Paris classification for disease extension (E4), and vitamin D level at baseline (T0) were the top predictors according to Logistic Regression model, while Random Forest’s most important features included vitamin D level at baseline (T0), Fecal calprotectin levels at baseline (T0), and Paris classification for disease extension (L2). Furthermore, robust computational diagnostics provided by both models enabled customized vitamin D adjustments with AUC values of 0.65 and 0.71 for remission prediction in Logistic Regression and Random Forest, respectively [21,22,23].

The predictive model’s performance is significantly impacted by inherent limitations of the dataset. The relatively small sample size may compromise statistical power and generalizability of results, leading to increased variability in parameter estimates. Furthermore, class imbalance, particularly underrepresentation of ‘Mild’ (1 patient) and ‘Severe’ (1 patient) severities, introduces bias, causing the model to favor majority classes while underperforming on minority classes, as indicated by low F1-Scores (0). Consequently, the stability of the model’s performance, especially in real-world scenarios with diverse class distributions, may be precarious, necessitating additional validation on larger and more balanced datasets to ensure robustness.

According to the results, young IBD patients undergoing complex therapies should receive 2000 IU of vitamin D_3_ as regular therapy. To reach serum levels exceeding 50 ng/mL, which are associated with peak remission rates, frequent monitoring of 25(OH)D and inflammatory markers is recommended due to substantial associations between vitamin D insufficiency and disease activity as well as prediction model performance [11,12].

Several alterations in vitamin D metabolism are age-related. Calcium absorption from the gut declines with age [24]. Individuals with normal vitamin D levels will experience these aging-related processes at a slower rate, resulting in a slower rate of ageing and better protection against age-related disorders [25].

Supplementing with 1000 IU per day increased vitamin D levels to the recommended range (above 75 nmol/L), but levels fell below this range following a 30-day break. Even after the break, a daily intake of 2000 IU kept vitamin D levels in the acceptable range [26].

Concentrations should exceed 30 ng/mL, since 25% of the US population and 60% of Central Europeans had levels below 20 ng/mL. This may be accomplished by taking 2000 IU (50 mcg) of vitamin D_3_ every day, which prevents diseases and death [27].

When feasible, supplements should be taken on a daily basis. Children who are vitamin D-deficient benefit the most from a daily dosage of 2000 IU of vitamin D_3_ [28]. When daily forms are not available or covered, ref. [29] recommend using intermittent dosing with the smallest available dose (≤50,000 IU) and the shortest interval between doses, as adequate daily medications (pills or soft capsules of 1000–2000 IU) are available. Supplementing with more than 2000 IU of vitamin D each day may help minimize the risk of gestational diabetes. Lower vitamin D supplementation (≤2000 IU/day) effectively reduced the risk of preeclampsia, with no significant difference compared to larger doses [30].

Vitamin D deficiency in IBD patients has been linked to a variety of adverse clinical outcomes, including increased disease activity, poor treatment response, higher recurrence rates, and an increased risk of both intestinal and extraintestinal consequences. Emerging methods emphasize the need of personalized therapies using precision nutrition initiatives. These include tailoring supplementation procedures to genetic polymorphisms impacting vitamin D metabolism, individual microbiome profiles, and particular disease phenotypes. Precision-based techniques seek to maximize treatment efficacy while minimizing potential risks associated with excessive or insufficient dosage [31].

In terms of therapy, ML models might be utilized as non-invasive decision-support resources to help clinicians identify patients at risk of losing remission before severe clinical changes occur [32]. A more proactive, data-driven strategy to managing IBD may result from the use of such high-precision algorithms, with treatment interventions like vitamin D_3_ supplementation optimized based on personalized prediction profiles [33].

IBD in adolescents and young adults is often associated with vitamin D deficiency, which correlates with the severity of the condition. Studies have shown that ML models can accurately classify pediatric IBD risk using serum 25(OH)D levels. However, there are no existing studies that directly predict responses to varying doses of vitamin D_3_ supplementation in this population. The development of hybrid mechanistic quantitative systems pharmacology-machine learning (QSP-ML) frameworks has the potential to simulate IBD clinical scores from inflammation biomarkers [34]. These models can provide a blueprint for optimizing dosage regimens, which could be further extended by incorporating pharmacokinetic data on vitamin D_3_ dosing. This personalized approach may help reduce flares in adolescents and young adults with IBD [12,35,36,37].

The ML models developed in this study (Random Forest and Logistic Regression) were optimized as classification algorithms to predict disease severity categories at follow-up. However, their architecture allows extension towards predicting future quantitative levels of biomarkers such as vitamin D, CRP, and calprotectin. By transitioning from a classification methodology to regression algorithms (e.g., Random Forest Regressor), it is theoretically possible to achieve precise predictions for these biomarker levels. This could be achieved by training the model with various combinations of independent variables at baseline (e.g., initial biomarker levels, 1000 IU/day vs. 2000 IU/day cholecalciferol dosages, disease phenotype, and other parameters). Exploring such regression models may provide clinicians with an advanced tool to simulate diverse therapeutic scenarios and proactively adjust doses, a direction we consider promising for future studies [38]. XAI provides insights into how a model arrives at its predictions or classifications, making it more transparent and trustworthy. Additionally, new approaches and advancements open new research directions (computer-aided diagnosis, Transformer architecture) [39,40,41].

The main limitations of this unicentric investigation into vitamin D supplementation in pediatric patients are the constraints imposed by daily dosage limits to mitigate toxicity risks, the prolonged duration required for correcting deficiencies, and the inadequacy of dietary or sunlight exposure as standalone sources of sufficient intake. A significant limitation of ML approaches for predicting vitamin D_3_ treatment response in adolescents and young adults with IBD is the limited size of available datasets. Future studies should prioritize collecting larger, multicenter datasets with prospective longitudinal tracking to enhance predictive robustness.

## 5. Conclusions

The association between vitamin D insufficiency and poor clinical outcomes in patients with established IBD underscores the significance of this micronutrient as a potential biomarker of disease severity [42]. The findings suggest that age-related factors, such as molecular mechanisms like vitamin D, play a crucial role in optimizing adolescents and young adults’ IBD therapy regimens. Notably, our results support the hypothesis that a 2000 IU dosage of vitamin D_3_ is more beneficial than 1000 IU in generating and sustaining remission in adolescents and young adults with IBD. The negative correlation between vitamin D_3_ levels and fecal calprotectin implies a direct anti-inflammatory action on the intestinal mucosa, further emphasizing the immunomodulatory effects of vitamin D. This study highlights the positive impact of high-dose vitamin D supplementation when used alongside standard treatment, leading to reduced disease severity in adolescents with IBD. Furthermore, our ML models’ effectiveness demonstrates that incorporating biochemical assessments into a predictive monitoring system may enable physicians to anticipate disease activity and adapt supplemental medications accordingly.

## Figures and Tables

**Table 3:** Demographic and clinical characteristics analysis.

Characteristics	Group A 1000 IU of Cholecalciferol (Vitamin D_3_) (N = 40)	Group B 2000 IU of Cholecalciferol (Vitamin D_3_) (N = 40)	*p*-Value
Age ^(a)^	19.27 ± 3.03	19.07 ± 3.03	0.968
Gender			
F	20 (50%)	21 (52.5%)	0.823
M	20 (50%)	19 (47.5%)	0.823
Disease			
Crohn disease	17 (42.5%)	15 (37.5%)	0.648
Ulcerative Colitis	23 (57.5%)	25 (62.5%)	0.648
Score			
PCDAI	17 (42.5%)	15 (37.5%)	0.648
PUCAI	23 (57.5%)	25 (62.5%)	0.648
Paris classification for disease extension			
E1	5 (12.5%)	4 (10.0%)	0.898
E2	4 (10.0%)	6 (15.0%)	0.898
E3	7 (17.5%)	5 (12.5%)	0.898
E4	7 (17.5%)	10 (25.0%)	0.898
L2	8 (20.0%)	8 (20.0%)	0.898
L3	9 (22.5%)	7 (17.5%)	0.898
Regimens used ^(b)^			
AS	4 (10.0%)	3 (7.5%)	0.989
AS, S	6 (15.0%)	5 (12.5%)	0.989
AS, S, ATB	2 (5.0%)	4 (10.0%)	0.989
AS, S, T	5 (12.5%)	6 (15.0%)	0.989
AS, S, T, ATB	4 (10.0%)	2 (5.0%)	0.989
S, T	8 (20.00%)	9 (22.5%)	0.989
S, T, ATB	5 (12.5%)	4 (10.0%)	0.989
T, anti-TNF	4 (10.0%)	5 (12.5%)	0.989
T, anti-TNF, ATB	2 (5.0%)	2 (5.0%)	0.989

^(a)^ Mean ± SD; ^(b)^ Regimen used: Amino-salicylates (AS), Steroids (S), Thiopurines (T), Antibiotics (ATB), anti–Tumor Necrosis Factor (TNF); A *p*-value < 0.05 was considered statistically significant.

**Table 4:** Statistical tests at baseline (T0).

Variable	Test	Statistics	*p*-Value
Age	T-Student	0.294	0.7690
Vitamin D	T-Student	0.070	0.9444
CRP	Mann–Whitney	644.00	0.1333
Fecal calprotectin	Mann–Whitney	789.50	0.9233
Score value	Mann–Whitney	800.00	1.0000

*p*-value refers to the between-group comparison at baseline (T0). A *p*-value < 0.05 was considered statistically significant. *p*-value refers to the between-group comparison at baseline (T0). Score value corresponds to PUCAI or PCDAI.

**Table 5:** ANOVA results.

Variable	Effect (Source)	F	*p*-Value	$\eta_{p}^{2}$
Vitamin D	Group	114.320	0.0000	0.594
	Time	258.249	0.0000	0.768
	Interaction	50.590	0.0000	0.393
CRP	Group	28.795	0.0000	0.269
	Time	86.316	0.0000	0.525
	Interaction	19.523	0.0000	0.200
Calprotectin	Group	46.575	0.0000	0.373
	Time	39.918	0.0000	0.338
	Interaction	24.293	0.0000	0.237

A *p*-value < 0.05 was considered statistically significant. If F > 1, this indicates that variability between groups exceeds within-group variability, reflecting the effect of the studied factor. $\eta_{p}^{2}$—effect size.

**Table 6:** LMM results.

Variable	Group A–Group B Time	Coef.	Std. Err.	z	*p* > |z|	Lower CI [95%]	Upper CI [95%]
Vitamin D	T1	12.57	2.36	5.32	0.0000	7.94	17.20
	T2	23.75	2.36	10.05	0.0000	19.12	28.38
CRP	T1	−20.85	5.74	−3.62	0.0000	−32.11	−9.58
	T2	−35.75	5.74	−6.22	0.0000	−47.01	−24.48
Calprotectin	T1	−61.75	20.72	−2.97	0.0030	−102.37	−21.12
	T2	−144.00	20.72	−6.94	0.0000	−184.62	−103.37

A *p*-value < 0.05 was considered statistically significant. CI—confidence interval 95%; z—z-score; Std. Err.—the uncertainty (lower is better); Coef.—The coefficient shows the effect size, how much the value increased (+) or decreased (−) compared to the reference group (Group A).

**Table 7:** Disease severity by group and disease.

Group	Severity	CD	UC	Total
Group A		17	23	40
	Remission	8	13	21
	Mild	1	0	1
	Moderate	8	9	17
	Severe	0	1	1
Group B		15	25	40
	Remission	13	22	35
	Mild	0	0	0
	Moderate	2	3	5
	Severe	0	0	0
Total		32	48	80

Crohn’s Disease (CD); Ulcerative colitis (UC); Total—patients’ distribution per each severity class for each group (A or B).

**Table 8:** Disease severity by group.

Disease Severity	Group A 1000 IU of Cholecalciferol (Vitamin D_3_) (N = 40)	Group B 2000 IU of Cholecalciferol (Vitamin D_3_) (N = 40)	*p*-Value
Remission	21 (52.5%)	35 (87.5%)	0.007
Mild	1 (2.5%)	0 (0.0%)	0.007
Moderate	17 (42.5%)	5 (12.5%)	0.007
Severe	1 (2.5%)	0 (0.0%)	0.007

Disease severity post-treatment (T2), a *p*-value < 0.05 was considered statistically significant.

**Table 9:** Vitamin D and inflammatory markers at baseline, after 3 months, and 6 months of treatment.

Vitamin D and Inflammatory Markers		Group A (N = 40)	Group B (N = 40)	*p*-Value
Vitamin D ^(a)^	Baseline (T0)	21.92 ± 5.01	21.85 ± 4.56	<0.001
	After 3 months (T1)	35.02 ± 11.15	47.52 ± 8.51	<0.001
	After 6 months (T2)	35.82 ± 8.51	59.50 ± 7.73	<0.001
CRP ^(b)^	Baseline (T0)	7.40 ± 4.25	10.50 ± 7.72	<0.001
	After 3 months (T1)	40.02 ± 22.73	22.27 ± 11.60	<0.001
	After 6 months (T2)	62.82 ± 37.14	30.17 ± 15.74	<0.001
Fecal calprotectin ^(c)^	Baseline (T0)	66.45 ± 37.05	66.00 ± 36.81	<0.001
	After 3 months (T1)	151.75 ± 84.33	89.55 ± 51.95	<0.001
	After 6 months (T2)	230.55 ± 110.35	86.10 ± 68.90	<0.001

^(a)^ ng/mL, ^(b)^ mg/L, ^(c)^ µg/g. Paired Samples *t*-test: Student’s *t*-test in Jasp version 0.19.3. *p*-value refers to within-group longitudinal change (T0, T1, T2). A *p*-value < 0.05 was considered statistically significant.

**Table 10:** Performance of ML models.

Model	Disease Severity (T2)	Precision	Recall	F1-Score	Patients
Logistic Regression	Remission	0.7931	0.8214	0.8070	56
	Mild	0.0000	0.0000	0.0000	1
	Moderate	0.5455	0.5455	0.5455	22
	Severe	0.0000	0.0000	0.0000	1
	macro avg	0.3346	0.3417	0.3381	80
	weighted avg	0.7052	0.7250	0.7149	80
	accuracy		0.7250		
Random Forest	Remission	0.8500	0.9107	0.8793	56
	Mild	0.0000	0.0000	0.0000	1
	Moderate	0.7500	0.6818	0.7143	22
	Severe	0.0000	0.0000	0.0000	1
	macro avg	0.4000	0.3981	0.3984	80
	weighted avg	0.8012	0.8250	0.8119	80
	accuracy		0.8250		

avg—average; Disease severity (T2) after 6 months. Performance metrics: precision, recall, F1-score for each model (Logistic regression or Random Forest).

**Table 11:** Logistic regression odds ratios.

Feature	Reference Class	Mild	Moderate	Remission
Vitamin D (T0)	Severe	0.9068	1.6355	0.7722
Age	Severe	1.0481	0.9130	0.9797
CRP (T0)	Severe	1.0037	0.9375	1.0552
Disease_Ulcerative Colitis	Severe	0.9260	1.0975	0.9207
Fecal calprotectin (T0)	Severe	0.9796	1.0442	1.0541
Gender_M	Severe	0.9122	1.0381	0.9589
Group_Group B	Severe	0.9321	0.6340	1.8002
Paris classification for disease extension_E2	Severe	0.9772	1.1091	0.9499
Paris classification for disease extension_E3	Severe	0.9693	0.8573	1.2479
Paris classification for disease extension_E4	Severe	0.9813	0.5846	1.4697
Paris classification for disease extension_L2	Severe	1.1533	0.7540	1.2025
Paris classification for disease extension_L3	Severe	0.9527	1.1834	0.9202
Score value (T0)	Severe	1.0523	1.2640	0.8421
Treatment_AS, S	Severe	0.9717	1.0547	1.0098
Treatment_AS, S, ATB	Severe	0.9778	1.1420	0.9229
Treatment_AS, S, T	Severe	0.9758	1.0436	1.0127
Treatment_AS, S, T, ATB	Severe	0.9856	0.7029	1.0741
Treatment_S, T	Severe	1.1380	1.1802	0.7818
Treatment_S, T, ATB	Severe	0.9718	0.8450	1.2474
Treatment_T, anti-TNF, ATB	Severe	0.9823	0.9200	1.1229
Treatment_T, anti-TNF	Severe	0.9889	0.7993	1.2883

Odds Ratios (each compared against ‘Severe’ class); Regimen used: Amino-salicylates (AS), Steroids (S), Thiopurines (T), Antibiotics (ATB), anti–Tumor Necrosis Factor (TNF); T0—baseline.

**Table 12:** Longitudinal safety parameters and renal function monitoring.

Parameter	Group A (1000 IU) T0	Group A (1000 IU) T1	Group A (1000 IU) T2	Group B (2000 IU) T0	Group B (2000 IU) T1	Group B (2000 IU) T2	*p*-Value *
Serum Calcium (mg/dL)	9.2 ± 0.4	9.3 ± 0.3	9.4 ± 0.4	9.1 ± 0.5	9.4 ± 0.4	9.5 ± 0.3	0.42
Urinary Calcium (mg/24 h)	165 ± 25	178 ± 30	185 ± 22	170 ± 28	192 ± 35	205 ± 28	0.15
Serum Creatinine (mg/dL)	0.82 ± 0.1	0.84 ± 0.1	0.81 ± 0.1	0.85 ± 0.1	0.83 ± 0.1	0.84 ± 0.1	0.88
eGFR (mL/min/1.73 m^2^)	102 ± 8	98 ± 10	104 ± 7	99 ± 9	101 ± 7	103 ± 8	0.65

* Data are presented as Mean ± SD. *p*-values refer to the between-group comparison at T2 using an independent samples *t*-test; *p*-value < 0.05 was considered statistically significant; eGFR (estimated Glomerular Filtration Rate).

## Data Availability

The data presented in this study are available on request from the corresponding authors. The data are not publicly available due to privacy and ethical restrictions.
